# Exploring initiation process and cultural identity towards young adult vapers in China: a qualitative comparison between single and dual users

**DOI:** 10.3389/fpsyg.2025.1480898

**Published:** 2025-03-20

**Authors:** Jingzhong Xie, Keda Wu

**Affiliations:** ^1^School of Journalism and Culture Communication, Zhongnan University of Economic and Law, Wuhan, China; ^2^School of Innovation, Hubei Institute of Fine Arts, Wuhan, China

**Keywords:** youth, e-cigarette, initiation process, temporal orientation, vaping

## Abstract

**Background:**

E-cigarette communication with conflicting views significantly shapes young people’s knowledge and attitudes, which are precursor predictors of their usage behavior. This study aims to explore the initiation process and perspectives on e-cigarettes from young adult vapers in China, especially in terms of culture.

**Method:**

In-depth one-on-one personal interviews and focus groups with 47 young adult vapers in China were conducted to understand their e-cigarette usage behavior and cultural understanding. Thematic analysis was employed to identify themes related to their initiation process and the cultural identity of e-cigarette use.

**Result:**

Three themes emerged regarding e-cigarette usage behavior among the young adult were identified: (i) e-cigarette initiation process and use pattern, (ii) temporal orientations towards risks and benefits, and (iii) self-construction of individual and relationship. The latter two themes reflect the cultural understanding that young adult vapers hold about e-cigarettes.

**Conclusion:**

Our qualitative evidence suggests that while single and dual users share a similar initiation process in general, their behavioral structures differ in significant ways in detail. Cultural factors such as time orientation and self-construction are crucial for tailoring health messages and designing interventions aimed at reducing e-cigarette use and addressing addiction among young adult vapers.

## 1 Introduction

E-cigarettes, introduced globally as a low-risk alternative to traditional cigarettes and as a potential smoking cessation aid (Hajek et al., 2014), have rapidly gained popularity. Due to their easy concealment, high nicotine content, and wide range of available flavors, e-cigarettes are particularly appealing to young users (King et al., 2018). Nationally representative cross-sectional data from the American Health Interview Surveys (2019–2021) show an increase in e-cigarette use among young people, with usage rising from 8.8 to 10.2% over the three-year period (Bandi et al., 2023). In China, the 2021 National College Student Tobacco Epidemic Survey conducted by the Centres for Disease Control and Prevention (CDC) found that 10% of college students have used e-cigarettes, with 2.5% currently using them (People’s Daily Online, 2022). These survey data and research evidence have sparked significant debate within the public health community regarding the potential harms and benefits of e-cigarettes.

Dual use of e-cigarettes and traditional tobacco products is common among young adults. Several prospective studies have shown that e-cigarettes may help reduce smoking or serve as a cessation aid (Caponnetto et al., 2013), which is promising for public health, as dual use could be part of a cessation trajectory for traditional tobacco products, thereby reducing the overall burden of tobacco-related diseases. However, some public health professionals express concerns that e-cigarettes may encourage young people to initiate smoking, prolong tobacco use among smokers attempting to quit, and undermine the effectiveness of anti-tobacco policies (Carroll et al., 2013; Regan et al., 2013). Therefore, gaining a comprehensive understanding of dual use is crucial for assessing the public health impact of e-cigarettes and developing targeted tobacco control interventions.

Considering the characteristics of the target is crucial when developing health campaigns aimed at effectively influencing public attitudes and behaviors (Hyman and Sheatsley, 1947), such as patterns of e-cigarette use and understanding vaping behavior. Researchers have approached the study of e-cigarettes and their antecedents and consequences from various angles, including the development of the product itself (Dusautoir et al., 2021), prevalence rates in the general population and demographic factors (Dai and Leventhal, 2019), users’ perceptions and motivations (Laverty et al., 2016; Smith et al., 2019; Dugas et al., 2020), and the relationship between e-cigarette use and relevant smoking trajectories (Hajek, 2013; Soneji et al., 2017). Very little research, however, has addressed the patterns and initiation process of e-cigarette use, particularly in comparison to the dual use of e-cigarettes and traditional tobacco products.

In health communication, culture is defined as a shared system of meanings, encompassing the values, beliefs, norms, practices, and communication patterns of a group with regard to health (Betancourt and López, 1993; Hughes et al., 1993). Previous studies showed that the specific cultural characteristics of a group can be directly or indirectly linked to health-related priorities, decisions, behaviors, and the reception of information in health education and interventions (Pasick et al., 1996). Despite widespread recognition of culture as an influential factor in health and behavior, it is often overlooked or misunderstood in related research, primarily due to the lack of clear operational definitions, metrics, and explanatory models (Kreuter and McClure, 2004). To address these gaps, scholars have developed theoretical models of health behavior, such as the PEN-3 cultural model (Iwelunmor et al., 2014) and culture-centered approach (Sastry et al., 2021), which demonstrate that culture plays a critical role in shaping an individual’s understanding of health and illness by influencing health perceptions and practices (Airhihenbuwa, 2007; Shaw et al., 2009). Therefore, understanding the broader cultural context in which a group lives is essential for a more nuanced interpretation of health behaviors and, consequently, the delivery of more effective, culturally congruent public health interventions.

Narratives have long been utilized to understand individual health experiences. These stories can deepen practitioners’ and researchers’ understanding of patients’ experiences by providing “meaning, context, and perspective for patient prediction” (Greenhalgh, 1999), serving as a vital theoretical and methodological tool for exploring health-related issues (Tang and Bie, 2016). In this regard, narratives offer valuable insight into the interplay between micro-level personal experiences and macro-level cultural values and ideologies, revealing individuals’ specific cultural interpretations of illness and health (Mattingly and Garro, 2000).

The current research conceptualizes e-cigarette usage as an interactive process, examining the causes, contexts, and subsequent outcomes for young adult vapers, and tracing the entire trajectory of usage. This study aims to explore two research questions by collecting narratives from young adult vapers in China: (1) What are the initiation processes and usage patterns of young adult vapers? (2) How do they perceive e-cigarettes and their usage behaviors, particularly within a cultural context? Additionally, the study specifically examines the distinctions between single and dual e-cigarette users. Our results will inform and advise improved estimates of the public health impact of dual use of e-cigarettes and the development of targeted interventions for tobacco control in young adults.

## 2 Method

### 2.1 Recruitment and data collection

After receiving approval from the Ethics Committee of author’s affiliation, participants were recruited. Data were collected using two methods: one-on-one interviews and focus group interviews. The combination of these two methods allows for data triangulation, facilitating in-depth exploration, enhancing understanding of the study population and context, and increasing the credibility of the findings (Lambert and Loiselle, 2008).

First, participants for the one-on-one interviews were recruited through online platforms, such as Weibo and Rednote, where young adults actively engage in discussions about e-cigarettes. A recruitment notice instructed potential participants to complete an online screening form to assess eligibility for e-cigarette products in the past 30 days have access to an e-cigarette for personal use. In addition, following the one-on-one interviews, focus group interviews were conducted to validate the initial findings. Recruitment for the focus groups was managed by a tobacco control organization affiliated with a university in central China. Eligibility criteria for the focus groups were the same as for the one-on-one interviews. The interviews were conducted from March to April 2021. In total, 16 participants took part in the one-on-one interviews, and 31 participants were divided into four groups for the focus group interviews. Both the one-on-one interviews and focus group interviews were conducted separately by the author of this study.

The interview questions were pre-tested in a small pilot study, which helped ensure their clarity and relevance. Before the interview, all participants signed an informed consent form. The interviews focused on the following topics: (1) smoking history and current status, including the age of initiation and quantity of cigarettes smoked; (2) e-cigarette history and usage patterns, such as frequency and contexts of use; (3) perceptions of e-cigarettes, including their health effects; (4) views of family members and friends regarding e-cigarettes; and (5) sources of information on e-cigarettes, including topics and channels. Interviews were recorded and transcribed verbatim with participants’ consent. All interviews were conducted in Chinese. The interview conversation was translated into English, and a backward-translation technique was applied to ensure the accuracy of the translation (Ozolins et al., 2020).

Table 1 provides details of the 47 participants who contributed qualitative data to this study. The sample included more males (*n* = 29) than females (*n* = 18). For daily e-cigarette use, 76.6% of participants preferred the device of cartridge the most, and 38.3% preferred the flavor of only menthol or mint the most. Participants were categorized into two main groups: e-cigarette single users (*n* = 15) and dual users of e-cigarettes and traditional cigarettes (*n* = 32). E-cigarette single users are those who currently use e-cigarettes daily or occasionally or have used e-cigarettes in the past 30 days but have never used other tobacco products. Dual users are individuals who currently use both e-cigarettes and traditional cigarettes and have not used other tobacco products besides these.

**Table 1:** Participants information (*n* = 47).

	*n*	%
Gender		
Male	29	61.7
Female	18	38.3
Age (years)		
18–22	28	59.6
23–25	19	40.4
Education		
Bachelor	33	70.2
Master	10	21.3
Other	4	8.5
Duration use of e-cigarette		
1 months	2	4.3
2–6 months	9	19.2
7–12 months	12	25.5
1–3 years	16	34.0
More than 3 years	8	17.0
Device type most used		
Disposable	6	12.8
Cartridge	36	76.6
Tank or mod	5	10.6
Flavor most used		
Only tobacco	10	21.3
Only menthol or mint	18	38.3
Only sweet	8	17.0
Any combination of tobacco, menthol or mint, and sweet	11	23.4
Investment in e-cigarette consumption		
Less than ¥100	4	8.5
¥101–300	10	21.3
¥301–500	14	29.8
¥501–1,000	12	25.5
More than ¥1,000	7	14.9
Single users of e-cigarette		
Daily vapers	11	23.4
Non-daily vapers	4	8.5
Dual users of e-cigarettes and traditional cigarettes		
Daily dual users	8	17.0
Predominant smokers	10	21.3
Predominant vpaers	9	19.2
Non-daily dual users	5	10.6

Following Borland et al.' (2019) method of cross-classifying e-cigarette and cigarette use based on product usage frequency, we further divided the e-cigarette single and dual users into subcategories. The e-cigarette single users were classified into two groups: (1) daily vapers (*n* = 11) and (2) non-daily vapers (*n* = 14). The dual users were classified into four subgroups: (1) daily cigarette smokers and daily e-cigarette users (“daily dual users,” *n* = 8), (2) daily cigarette smokers and non-daily e-cigarette users (“predominant smokers,” *n* = 10), (3) non-daily cigarette smokers and daily e-cigarette users (“predominant vapers,” *n* = 9), and (4) non-daily users of both products (“non-daily dual users,” *n* = 5).

### 2.2 Data analysis

Data analysis for the one-on-one interviews and focus groups followed the inductive approach of thematic analysis. The authors employed open coding, axial coding, and selective coding to identify and generalize themes from the transcribed data (Strauss and Corbin, 1990). Initially, the transcribed data were open-coded to identify as many preliminary concepts related to e-cigarette use as possible through inspection, comparison, and classification. These concepts were then clustered into initial categories for axial coding based on their attributes and dimensions. Finally, the thematic structuring of the coding process clarified the underlying and cultural identities associated with young vapers’ e-cigarette acceptance and usage. Throughout the coding process, stages were continuously compared and integrated until no new concepts, categories, or themes emerged, indicating saturation (Strauss, 1987).

The coding process followed predefined guidelines to operationally define the codes to enhance reliability and validity. Two trained coders, independently coded a random sample from each of the individual interviews and focus groups to ensure consistency (k ≥ 0.8), enhancing the reliability of the coding process (Creswell and Poth, 2016). Any discrepancies or modifications regarding the coding guidelines were discussed and resolved throughout the process. During the coding process, the source of each participant (one-on-one interview or focus group) and their e-cigarette usage role (single user or dual user) were noted. For instance, “P01-S” indicates that the first participant in the one-on-one interview was a single user of e-cigarettes. “G02-4D” denotes the fourth participant in the second focus group, a dual user.

## 3 Results

The coding process yielded three primary themes and nine subthemes (see Table 2). The three primary themes identified are: (1) e-cigarette initiation process and usage patterns, (2) temporal orientations towards risks and benefits, and (3) self-construction of individuals and relationships. The latter two themes are particularly concerned with the cultural understanding of e-cigarettes. In the following section, we will illustrate these themes within the narratives of young adult vapers, analyzing the differences in narrative themes between single and dual users, with a focus on their respective roles in e-cigarette use.

**Table 2:** Summary of themes and subthemes.

Theme	Subtheme	Number	Representative quotes
E-cigarette initiation process and use pattern	1.1 Characters	18	“I only use e-cigarettes, it’s a personal thing for me.” (P01-S) “In my daily life, I smoke traditional cigarettes significantly more often than I smoke e-cigarettes. E-cigarettes can only be a substitute.” (G02-3D)
	1.2 Setting	12	“A classmate who smokes e-cigarettes recommended it to me, and I tried a few puffs with him in the bathroom.” (G02-5S) “People around me have switched from traditional cigarettes to e-cigarettes, so I followed suit.” (P09-D)
	1.3 Problem	9	“My roommate brought a new gadget that did not smell like smoke, it is novel.” (G03-4D) “Traditional cigarette users use e-cigarettes as an aid to quit smoking.” (G02-6D)
	1.4 Actions	15	“I feel tight and bloated in my chest after smoking e-cigarettes.” (P14-D) “I smoke e-cigarettes a lot because it’s convenient. I can take it out anytime and anywhere and take a few puffs.” (G04-5D) “It tends to ‘plug the smoke’. Oil leaks are also a nuisance.” (G03-5D)
	1.5 Resolution	6	“The first time I tried smoking after feeling quite fun, and then I bought one myself. Gradually I began to smoke electronic cigarettes every day.” (G02-5S)
Temporal orientations to risk and benefit	2.1 Future-oriented risk avoidance	13	“E-cigarettes, as a new thing, still have many potential risks with the addition of many artificial chemical ingredients.” (P06-D)
	2.2 Present-focused benefit acquisition	10	“Now after using it, I find it is convenient, environmentally friendly and now most people can accept it.” (P15-S)
Self-construction of individual and relationship	3.1 Self-satisfaction dominated by personal needs	22	“Smoking e-cigarettes not only because it feels cool, but also feel very ‘comfortable’.” (G02-5S)
	3.2 Compliance with norms dominated by social relationships	19	“When I’m going to use an e-cigarette, I usually ask people if they mind. After all, I do not want to be disliked by others.” (G01-1D)

### 3.1 E-cigarette initiation process and use pattern

Our findings revealed notable differences in usage patterns and initiation processes between single users and dual users. To illustrate these variations, we created two schematic diagrams representing the acceptance and usage processes of e-cigarettes for each user category.

**Figure 1:**
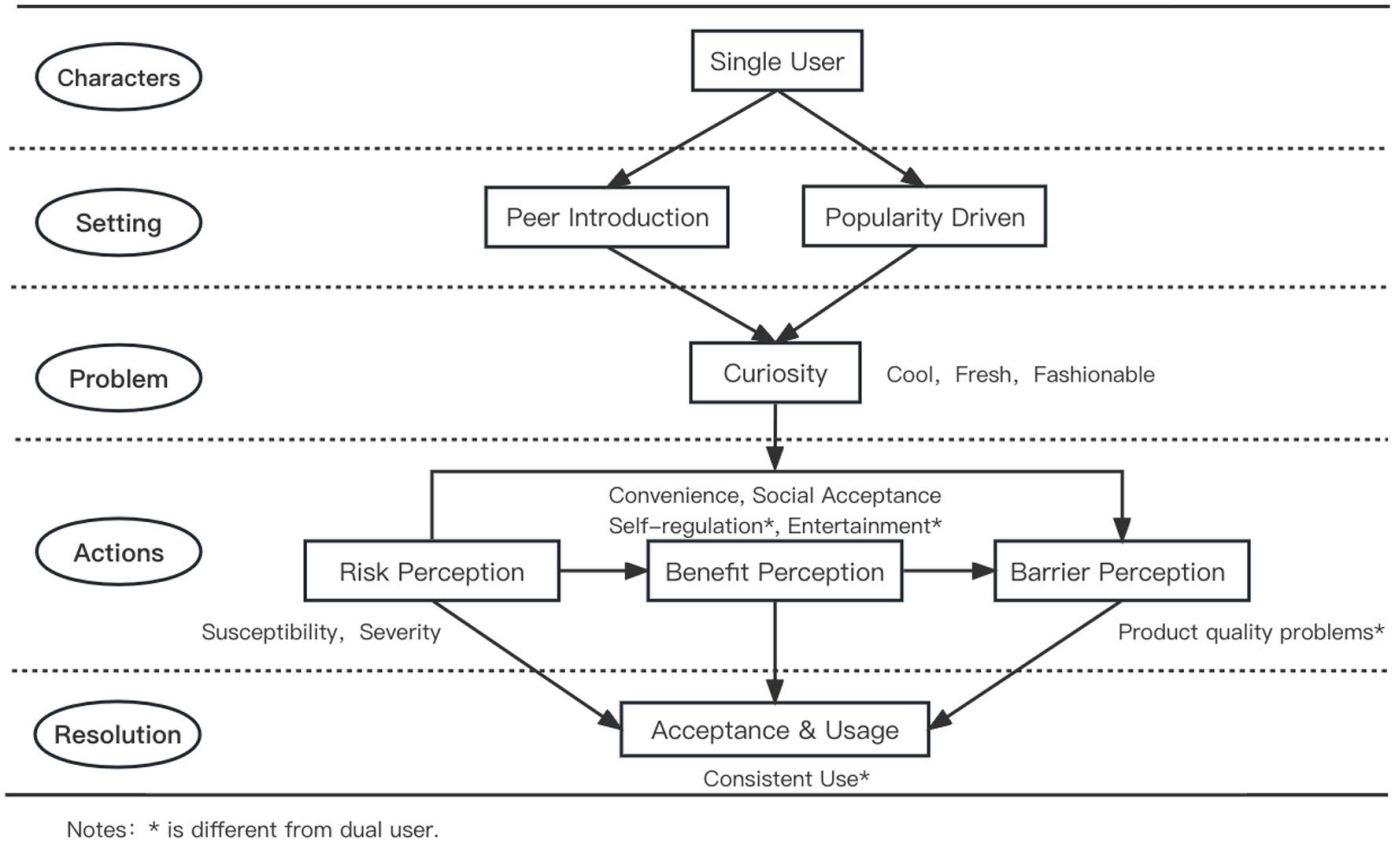
Initiation process of e-cigarettes for single users.

Figure 1 illustrates the initiation process of e-cigarettes for single users. As shown, single users are primarily introduced to e-cigarettes by peer’s influence, with the product’s popularity and social appeal playing a key role in their decision to try it. Single users perceive e-cigarettes as cool, novel, and trendy, driven by curiosity. Their initial focus is on the benefits related to self-regulation and entertainment, while they weigh potential barriers, such as product quality concerns. After a thorough assessment of the perceived risk perceptions, benefits, and barriers, they decide to accept and continue using e-cigarettes.

**Figure 2:**
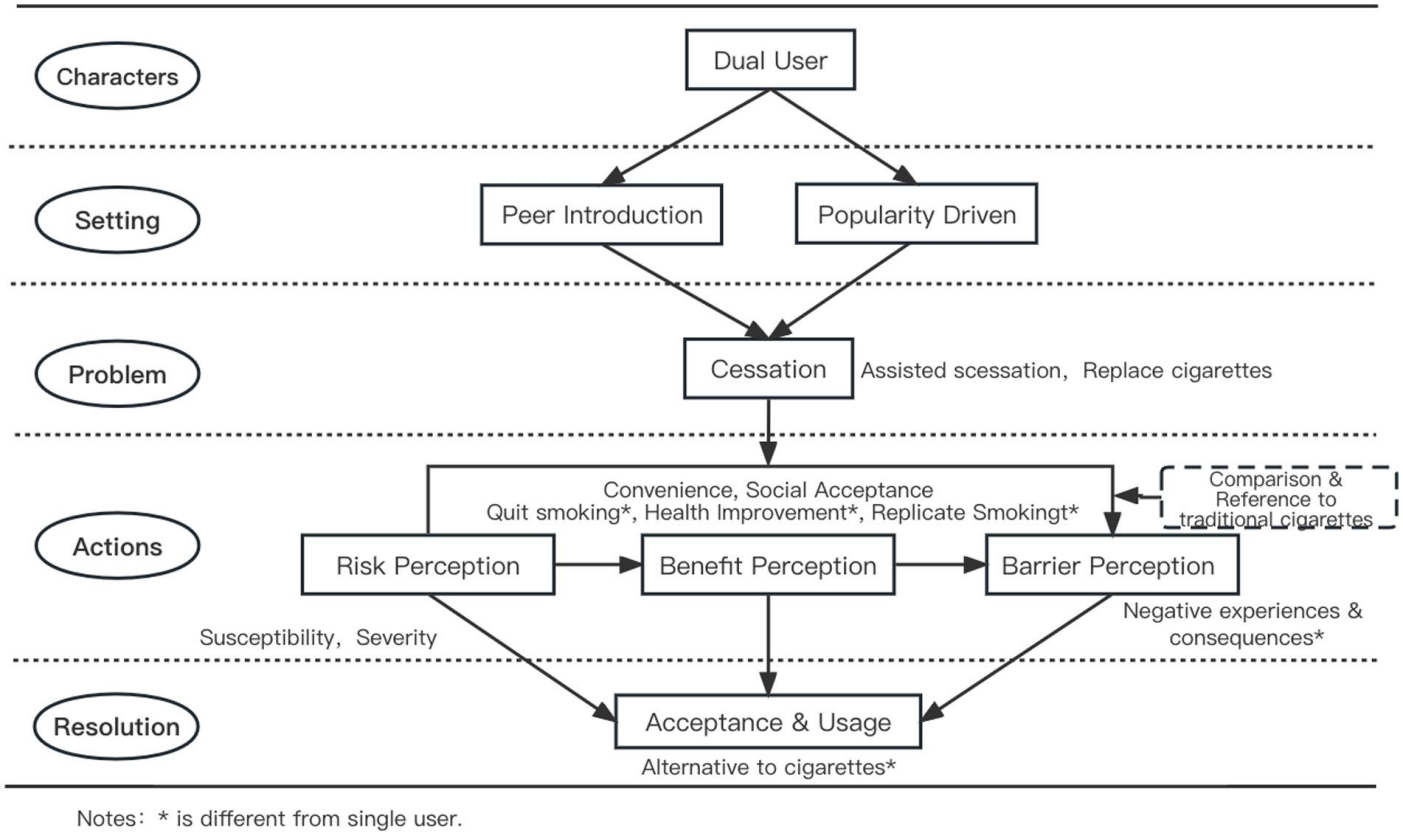
Initiation process of e-cigarettes for dual users.

Figure 2 depicts the initiation process for dual users. While the initial context for dual users’ introduction to e-cigarettes mirrors that of single users, dual users tend to view e-cigarettes as a tool for quitting traditional smoking. Among the participants, most young adult vapers had previously smoked traditional cigarettes and transitioned to dual use. Given their experience with traditional cigarettes, dual users frequently compare e-cigarettes to their prior habits, with a strong emphasis on the perceived benefits of smoking cessation and health improvement. They also evaluate barriers such as negative e-cigarette experiences and potential health risks. After evaluating these factors, dual users either continue using e-cigarettes alongside traditional cigarettes or substitute them for conventional smoking in their daily routines.

### 3.2 Temporal orientation towards risks and benefits

Health behaviors often involve both immediate and delayed costs and benefits, making temporal orientation a critical factor in health decision-making. Cultural understanding of temporal orientation plays a significant role in shaping young adults’ experiences with e-cigarettes, particularly regarding two key dimensions: (i) future-oriented risk avoidance and (ii) present-focused benefit acquisition.

#### 3.2.1 Future-oriented risk avoidance

Individual differences in considering the short or long-term consequences of health behaviors reflect not only future orientation but also a cognitive mindset. This mindset, known as consideration of future consequences (CFC), refers to the extent to which individuals take into account the potential long-term outcomes of their current behaviors (Strathman et al., 1994). In the context of e-cigarettes, future-oriented risk avoidance has been observed in the responses of young adult vapers, who consider the potential long-term risks of e-cigarettes. As previously noted, e-cigarettes remain controversial in public health discourse due to their status as a novel tobacco product, with public awareness about their risks still evolving. As a result, young adult vapers tend to avoid potential future risks associated with e-cigarettes, which influences their long-term usage decisions. For instance, dual users—those who use both e-cigarettes and traditional cigarettes—are more likely to engage in risk comparisons between the two products. One participant, G02-4D, expressed the following concern:

“*E-cigarettes are and come with many potential risks. I do not want to take a chance and try something when I cannot predict the consequences…there are no worse outcomes about using traditional cigarettes, like my father’s generation.*”(G02-4D)

For dual users, prior experience with traditional cigarettes raises their baseline for risk perception baseline risk perception, leading them to approach e-cigarettes more cautiously. They are more likely to compare the unknown risks of e-cigarettes with the well-understood risks of traditional smoking, resulting in a reluctance to fully embrace e-cigarettes as a safer alternative. Consequently, they adopt an avoidance strategy regarding e-cigarettes, considering their potential future risks compared to traditional cigarettes. This is particularly true for dual users who predominantly smoke traditional cigarettes. In their daily dual use, they mainly rely on traditional cigarettes and turn to e-cigarettes only in contexts where smoking is restricted, driven by social norms surrounding smoking. One predominant smoker explained:

*“E-cigarettes do not taste as good. I still prefer the feeling traditional cigarettes give me, and I only use e-cigarettes occasionally, like when I’m in public places where smoking is not allowed. I vape to satisfy my cravings in those situations.”*

#### 3.2.2 Present-focused benefit acquisition

Temporal discounting refers to the tendency to devalue future rewards in favor of immediate ones, a phenomenon commonly observed in health-related decision-making (Chapman, 1996). Young adult vapers exhibit temporal discounting when evaluating e-cigarette use, prioritizing immediate benefits over long-term outcomes. Interviews revealed several attributes of e-cigarettes that attract youth, with a particular emphasis on the benefits. These benefits include sensory gratification, discreet use, low cost, and convenience. Most of these benefits are immediate consequences of e-cigarette use, which fulfill various needs of young adult vapers and encourage continued use. As a single user explained:

“*The smoke of e-cigarette will not be rejected by all. Some people even like the light flavor. I can take a puff anytime, in many places – it’s so convenient*.” (G04-3S)

Single users, unlike dual users, lack experience with traditional cigarettes and are unfamiliar with the health risks and negative experiences associated with smoking. For them, immediate benefits—such as rich flavors, a cool appearance, greater social acceptance, and convenience—are the primary motivations for using e-cigarettes. Additionally, the widespread prevalence of e-cigarettes in media and advertising plays a significant role in attracting young adults to try them. This is especially true for non-daily smokers, who are more likely to be influenced by marketing and start using e-cigarettes for emotional factors such as enjoyment (Saddleson et al., 2016). As one non-daily vaper shared:

*“The amount of e-cigarette advertisements I see online (on social media, news sites) and offline (in stores, shopping malls, tobacco specialty shops) really piqued my interest. My close friends are using them too.”* (P12-S)

### 3.3 Self-construction of individual and relationship

Self-construction is a concept that explores how individuals perceive themselves in relation to others and how they represent themselves in social contexts (Banaji and Prentice, 1994). It originated from the comparison of individualistic and collectivistic cultural dimensions (Hofstede, 2011). Based on these dimensions, self-construction can be categorized into two distinct types: independent self-construction and interdependent self-construction (Markus and Kitayama, 1991). As a reflection of cultural orientations, self-construction can help explain social behavior and examine cultural differences between societies (Levine et al., 2003). In the context of e-cigarette use among young adult vapers, self-construction influences their behavior in two ways: (i) Self-satisfaction dominated by personal need and (ii) Compliance with norms dominated by social relationship.

#### 3.3.1 Self-satisfaction dominated by personal need

Individuals with independent self-construction tend to focus on their internal attributes, with a strong emphasis on personal goals and interests (Cross et al., 2011). They distinguish themselves from others, by emphasizing their unique personal attributes in public settings (Matsumoto and Juang, 2016). Young adult vapers who exhibit independent self-construction use e-cigarettes to satisfy their individual needs. For them, the immediate benefits—such as relieving cravings and enjoying the novel experience of vaping – are key motivations. While they are influenced by the social norms of family and friends (Hanafin et al., 2021), this influence is limited, and they tend to persist in their e-cigarette use despite opposition from family members. A single user described his experience with e-cigarettes:

*“…after vaping, it feels like floating in the clouds… When the craving comes, I can take a puff any time and anywhere, like when playing games, in the bathroom, or in bed… Even though my parents have reprimanded me several times, I still use them secretly.”*(P10-S)

The self-construction of independence is evident in the e-cigarette use patterns of single users. Unlike traditional cigarettes, e-cigarettes lack the social attributes often associated with smoking, as each vaper typically owns a personalized device that is not commonly shared. As a result, e-cigarette use in China tends to be a one-person behavior rather than a collective or social activity. With the increasing social acceptance of e-cigarette flavors (Hung et al., 2022), single users are increasingly less likely to be influenced by environmental constraints and instead focus more on fulfilling their personal needs. This is particularly true for daily vapers, who tend to use e-cigarettes more frequently and spend more on them than other types of users. As one daily vaper shared:

*“My spending on e-cigarettes has increased substantially due to the unrestricted use, and it’s causing me financial pressure.”* (G02-5S)

#### 3.3.2 Compliance with norms dominated by social relationship

Individuals with interdependent self-construction typically foster harmony among group members through the development of close relationships (Cross et al., 2011). They are more attentive to each other’s views and opinions, adjusting their behaviors accordingly (Matsumoto and Juang, 2016). Similarly, young adult vapers adhere to social norms when vaping or smoking to align with the group, reflecting a collectivist self-construction. They emphasize rational cognition and judgment regarding e-cigarettes, and prioritize long-term, collective interests. One dual user, for instance, mentioned:

“*Some scientific information believes that long-term use of ecigarettes can also be harmful to the body, but less than traditional cigarettes.*”(P16-D)

In addition, dual users are acutely aware of social relationships during their use of e-cigarettes and traditional cigarettes. As mentioned earlier, dual users compare e-cigarettes to traditional cigarettes not only in terms of potential health risks but also regarding the inhibitions and social norms surrounding their use. They are influenced by social expectations, particularly concerns about secondhand smoke and the general acceptance of e-cigarettes. With the growing consensus that smoking is harmful to health, including the risks of secondhand smoke, more smokers are turning to e-cigarettes to avoid smoking-related illnesses and minimize peripheral effects, ultimately seeking better integration into social groups. As one dual user who predominantly uses e-cigarettes shared:

*“I use traditional cigarettes only occasionally because they are not allowed in many places. Even when I use e-cigarettes, I usually check with those around me to see if I’m bothering anyone.”* (P07-D)

## 4 Discussion

This study explored the perspectives of young adult vapers regarding e-cigarettes, focusing on their acceptance processes and usage behaviors. The analysis revealed distinct initiation processes between single users (only e-cigarette) and dual users (both e-cigarette and traditional cigarettes), as well as unique cultural identities shaped by temporal orientation and self-construction.

While both single users and dual users exhibit similar overall usage patterns, they differ significantly in the detailed behavioral structures of their e-cigarette initiation. Initially, both groups are introduced to e-cigarettes through peer recommendations or popular trends, and they evaluate the risks benefits, and barriers before making decisions. However, their motivations differ: single users are primarily driven by curiosity, while dual users are motivated by a desire to quit smoking. Dual users, with prior experience in traditional cigarettes, consistently compare e-cigarettes with traditional cigarettes when evaluating their use. The findings align with existing literature on the reasons and contexts of e-cigarette use among young adults (Hummel et al., 2015; Smith et al., 2019).

Moreover, the study found that dual users tend to hold less favorable attitudes toward e-cigarettes compared to single users, perceiving fewer risks associated with their use (Farsalinos et al., 2015; Romijnders et al., 2018). This suggests that dual users’ perceptions of e-cigarettes are significantly influenced by their prior smoking experience. When e-cigarettes meet their expectations for quitting smoking, they continue to use e-cigarettes as a cessation aid, in contrast, they have used e-cigarettes as substitutes for traditional cigarettes, especially when access to traditional cigarettes is restricted. Consistent with the findings of Borland et al. (2019), predominantly vapers were less likely to report that vaping was less satisfying than smoking and were more likely to feel the benefits of quitting; whereas predominantly smokers mainly experimented with vaping and did not use nicotine e-cigarette products as an alternative to smoking, a similar finding was also seen in Yong et al.'s (2019) survey. These insights underline the critical role that motivation and prior smoking experience play in shaping e-cigarette usage behaviors. Therefore, when developing targeted health communication strategies for e-cigarette users, it is essential to account for these factors—particularly in distinguishing between users motivated by cessation versus those driven by curiosity or other social influences.

Temporal orientation emerged as a key factor distinguishing single users from dual users. Single users focus more on the immediate benefits of e-cigarettes, while dual users are more concerned with avoiding future risks. This is consistent with research on CFC, which shows that individuals with low CFC prioritize immediate needs and concerns, while those with high CFC focus on the future impact of their behavior and adjust their actions accordingly (Morison et al., 2010). Dual users, having experience with traditional cigarettes, bring a heightened awareness of smoking-related harms, which leads them to consider the future risks of e-cigarette use more carefully. As a novel product, e-cigarettes present uncertainties for dual users, further complicating their decision-making process. As previous studies have shown, some dual users are in torn between the use of e-cigarettes and traditional cigarettes, and they are still experimenting with vaping because they are still skeptical that the e-cigarette product is good enough to draw them away from smoking (Borland et al., 2019). Therefore, health communication directed at dual users should emphasize the potential uncertainties and risks of e-cigarettes, while messages for single users should highlight the immediate dangers, such as safety concerns and the declining popularity of e-cigarettes.

The study also explores the role of self-construction in e-cigarette use. Single users, with independent self-construction, prioritize personal satisfaction over the long-term consequences of their actions. In contrast, dual users exhibit more interdependent self-construction, focusing on the impact of their behavior on others and social norms related to traditional cigarette use. These findings align with previous research suggesting that e-cigarette-related interpersonal communication and perceived norms are associated with e-cigarette use (Awua et al., 2024). For dual users, social norms around smoking behavior are particularly salient, influencing their decisions regarding e-cigarette use. This is consistent with research showing that young adult vapers, particularly those aged 18–24, are more likely to stop using e-cigarettes due to public disapproval compared to older vapers (Yong et al., 2019). The heightened sensitivity to social judgment in this age group underscores the critical role of social norms in shaping e-cigarette use among young adults. Thus, interventions aiming to discourage e-cigarette use among this demographic can leverage social norms as a tool. Public health messages that emphasize the potential harms of e-cigarettes, coupled with promoting the social unacceptability of their use, may effectively reduce social acceptance and influence behaviors. By highlighting the collective consequences of e-cigarette use—both for the individual and for the broader community, such messages can appeal to the interdependent self-construction of dual users, reinforcing the importance of social responsibility and long-term health considerations.

## 5 Implications and limitations

Our study offers significant theoretical and practical implications. The effectiveness of message tailoring is significantly shaped by contextual factors, which necessitate formative research (Moskowitz et al., 2007) and, to some extent, the researcher’s cultural understanding of the target group (Maxwell et al., 2003). Although previous research have examined the antecedents and consequences of e-cigarette behavior, this study takes a step forward by integrating these factors into a more comprehensive framework that conceptualizes e-cigarette usage as an interactive process. By exploring the causes, contexts, and outcomes of behavior among young adult vapers, this study offers a nuanced understanding of the initiation and usage patterns of e-cigarettes. The comparison between single and dual users reveals distinct behavioral structures, shedding light on how each group’s motivations, experiences, and perceptions shape their consumption of e-cigarettes. Furthermore, the study highlights the cultural identities tied to e-cigarette use, with particular emphasis on temporal orientation and self-construction. Understanding these cultural factors enhances the ability to distinguish between different user groups, enabling the refinement of persuasive messaging in health communication campaigns and interventions.

This study also has several limitations. First, the sample was primarily composed of young adults, and therefore, the perspectives of adolescent vapers regarding e-cigarettes are not included. This limits the generalizability of the findings, making the results most relevant for health practice strategies targeted at young adults rather than the broader population of e-cigarette users. Second, while focus groups were employed as the primary data collection method, there is a potential for bias due to the group dynamics. Participants may have been influenced by the opinions and behaviors of others in the group, leading to social desirability bias or conformity. Finally, while we analyzed differences in the e-cigarette initiation process and cultural identity between single and dual users, differences in dual user subtypes need to be further examined in future qualitative studies as contributing to a more refined understanding of behaviors across different classifications of e-cigarette use.

## 6 Conclusion

The findings of this study underscore the importance of understanding the differing initiation processes of single and dual users in relation to e-cigarette consumption. These insights highlight how motivation and prior smoking experience are key factors that influence user behaviors and should be taken into account when developing health messages for each group. Furthermore, by identifying cultural dimensions such as temporal orientation and self-construction, the study contributes to a deeper understanding of how young adult vapers perceive and interact with e-cigarettes. These cultural factors are pivotal in shaping health behaviors and can be leveraged to design more effective behavioral interventions. The results also provide valuable guidance for future interventions, emphasizing the need for a more personalized, culturally sensitive approach to health communication. This ensures that e-cigarette use is effectively addressed across diverse segments of the population, ultimately leading to more successful public health strategies.

## Data Availability

The raw data supporting the conclusions of this article will be made available by the authors, without undue reservation.
